# Screening, production, optimization and characterization of β-glucosidase using microbes from shellfish waste

**DOI:** 10.1007/s13205-016-0530-7

**Published:** 2016-10-03

**Authors:** Samhita Mahapatra, A. S. Vickram, T. B. Sridharan, R. Parameswari, M. Ramesh Pathy

**Affiliations:** SBST, VIT University, Vellore, 632014 Tamil Nadu India

**Keywords:** Artificial neural networks, *β*-glucosidase, Plackett–Burman, *Proteus mirabilis*, Optimization, Response surface method

## Abstract

An extracellular *β*-glucosidase was isolated from *Proteus mirabilis* VIT117 found to be growing on prawn shells. The enzyme production was found to be enhanced (14.58 U/ml) when the culture was maintained at pH 9 and provided with sorbitol as carbon source, yeast extract as nitrogen source and incubated at 37 °C for approximately 72 h. Statistical methods like Plackett–Burman and RSM were also applied here to study the effects of different combinations of growth parameters for the bacteria, where the most significant parameters were found to be inoculum size, pH, yeast extract, incubation time and sorbitol. The optimum concentrations of inoculum size, pH and yeast extract determined by RSM were 2 %, 9 and 2 %, respectively. Partial purification of the protein was done by ammonium sulfate precipitation, followed by dialysis, gel filtration chromatography and SDS-PAGE. The enzyme was found to have a molecular weight of approximately 50 kDa and was observed to be most active at 37 °C in pH 9, with a sharp decline in the enzyme activity when temperature or the pH was increased. Enzyme kinetics study was performed to understand the catalytic behavior of the enzyme and it was found that our *β*-glucosidase had 5.613 U/ml and 0.082 mM as *V*_max_ and *K*_m_ values, respectively.

**Electronic supplementary material:**

The online version of this article (doi:10.1007/s13205-016-0530-7) contains supplementary material, which is available to authorized users.

## Introduction

*β*-glucosidase (E.C.3.2.1.21) comes from widespread sources including bacteria, fungi, animals, and plants. It exhibits well defined wide variety of substrate and simple nature of enzyme assay (Shewale 1982). It is capable of cleaving *β*-glucosidic linkages of conjugated glucosides and disaccharides. It acts upon β1→4 bonds linking two glucose or glucose-substituted molecules. It catalyzes the hydrolysis of β-glucosidic linkages in di- and oligo-glucosaccharides and several other glycols conjugate (Esen et al. 1993). It catalyzes the hydrolysis of terminal non-reducing residues in *β*-D-glucosides with release of glucose. Many of them also show synthetic activity via reverse hydrolysis or transglycosylation (Reeta et al. 2012). In cellulolytic microorganisms, *β*-glucosidase is involved in cellulase induction and cellulose hydrolysis (Bisaria and Mishra 1989; Tomme et al. 1995). In plants, the enzyme is involved in beta-glucan synthesis during cell wall development, pigment metabolism, fruit ripening, and defence mechanisms (Brozobohaty et al. 1993) whereas, in humans and other mammals, *β*-glucosidase is involved in the hydrolysis of glucosyl ceramides (Lieberman et al. 2007). So defects in *β*-glucosidase activity in humans are associated with Gaucher’s disease, a non neuropathic lysosomal storage disorder. It is a genetic disease where fatty substances like sphingolipids accumulate in cells and other organs. Hydrolysis of soybean isoflavone glycosides is an important application of *β*-glucosidase in industries. Due to their wide and varied roles in nature, these versatile enzymes can be of use in several synthetic reactions as reviewed by Bhatia et al. (2002). In recent years, interest in these enzymes has gained momentum owing to their biosynthetic abilities. Attempts are being made to understand the structure–function relationship of these versatile biocatalysts.

Depletion of fossil fuel at enhanced rate and its effect on global economic and environment has accelerated the research on alternative fuels like bioethanol. *β*-glucosidase is now used for the synthesis of biofuel. Enzymatic hydrolysis of cellulose is a multistep complex process, the last step being a homogenous catalysis reaction involving the action of *β*-glucosidase on cellobiose (Lynd et al. 2002). The structure is divided into lignin and hemicellulose. The cellulase can better access the structure to act on it. In the second step, hydrolysis of cellulose by cellulase takes place. Endoglucanase breaks the chain in the middle of the molecular structure of cellulose. Exoglucanase binds an available end of the chain and isolates it. Then units of cellobiose are cut (two units of glucose which are together). To finish, *β*-glucosidase divides cellobiose into two glucoses. When they ferment, they become ethanol. The final product is obtained by fermentation, distillation and dehydration. For industrial use, isolation and characterization of new high yielding strains of *β*-glucosidase using less costly carbon source still remains a challenge. The objective of this study was to isolate one such strain, to characterise it, and to experimentally find out the favourable conditions required for it to produce maximum amount of *β*-glucosidase, so that it could be used on an industrial scale.

## Materials and method

### Microorganism

Shellfish wastes (prawn shells gathered from local fish market, Vellore, Tamil Nadu, India) were used as the source for microorganisms producing *β*-glucosidase. Culturing was done in Luria Bertini broth. Out of 14 bacterial species isolated, the organism producing the maximum quantity of *β*-glucosidase among them was selected as the organism of interest. 16S rRNA sequencing was carried for the selected strain and BLAST results obtained from the 16S rRNA sequencing were used to find the percentage sequence similarity. Clustal W was used to construct a phylogenetic tree to identify the organism. A gram-negative biochemical test kit was used to identify the properties of the bacteria.

### Growth curve and *β*-glucosidase production

A growth curve was plotted where OD was checked at 600 nm, at an interval of 4 h approximately, staring from the time of inoculation. Readings were taken for the next 3 days from the time of inoculation. Bacterial cells obtained from culture were checked for the presence of intracellular as well as extracellular secretion of *β*-glucosidase. In case of extracellular enzyme secretions, culture broth was centrifuged at 8000 rpm for 10 min, after which the supernatant was collected for carrying out the enzyme assay. To check for intracellular enzyme secretions, the culture broth was subjected to ultrasonication with a pulse of 30 s for 3 min, followed by centrifugation at 8000 rpm for 10 min and the supernatant was collected for assay.

### *β*-glucosidase enzyme assay

A simple yet effective enzyme assay was carried out to check for *β*-glucosidase, according to Chang et al. (2011). To 1 ml of the supernatant obtained, 1 ml of 5 mM p-Nitrophenyl-β-D-glucopyranoside (pNPG) solution was added, which was then incubated at 45 °C for 10 min with shaking. The reaction was stopped by adding 1 ml of 2 M Na_2_CO_3_. The activity of *β*-glucosidase was estimated spectrophotometrically where absorbance was measured at 410 nm. The enzyme activity can be then determined from standard curve. Unit of enzyme activity is U/ml, where U is the amount of 1 mol of p-NP (para-nitrophenol) produced per minute at 37 °C.

### Effect of various parameters on *β*-glucosidase production

Effect of varying incubation time span was checked. Autoclaved LB broth inoculated with 2 % inoculum was checked for *β*-glucosidase activity after 12, 24, 36, 48, 60, 72, 84 and 90 h from the time of inoculation. The effect of five carbon sources (sucrose, fructose, tri-sodium citrate, sorbitol and starch) on the production of *β*-glucosidase was studied. Each carbon source was taken at 1 % concentration along with 2 % inoculum and incubated for 3 days at 37 °C, after which *β*-glucosidase assay was done to check enzyme activity. The effect of five nitrogen sources (urea, ammonium sulfate, peptone, yeast extract and glycine) on the production of *β*-glucosidase was studied. Each nitrogen source was taken at 1 % concentration along with 2 % inoculum and incubated for 3 days at 37 °C, after which *β*-glucosidase activity was measured by standard assay. pH of LB broth samples were adjusted to 4, 5, 6, 8, 9 and 10 and inoculated with 2 % inoculum and incubated at 37 °C for 3 days. *β*-glucosidase activity was checked using the standard assay. LB broth wAS inoculated with 2 % inoculum and incubated at temperatures 4, 15, 30, 37, 55, 60 and 85 °C for 3 days, after which activity of *β*-glucosidase was checked using standard assay.

### Plackett–Burman method

The most significant variables that effected the *β*-glucosidase production were studied by Plackett–Burman method. Nine main variables which included starch, sorbitol, yeast extract, ammonium sulfate, sodium chloride, tween 80, incubation time, pH, inoculum size were taken into consideration for the study along with two dummy variables. Based on Plackett–Burman factorial design, each variable was examined at two levels: −1 for low level and +1 for high level, and a centre point was run to evaluate the linear and curvature effects of the variables. *β*-glucosidase activity assay was carried out in triplicates and the average of these values was taken as response. The quality of the fit of the model equation was determined by the coefficient *R* ^2^ and the statistical significance by *F* test.

### Response surface methodology analysis

To locate the true optimum concentrations of significant variable for enzyme production, a Central composite design (CCD) with five coded level was conducted, in the subsequent phase of the statistical approach. This trial was conducted using a full 23 factorial design with six axial points and six replications of the centre point, resulting in a total number of 20 experiments. Using a second-order polynomial equation, *β*-glucosidase production was analyzed and fitted into the equation by the multiple regression procedure. The quality of fit of the second-order model equation was expressed by the coefficient *R* ^2^, and its statistical significance was determined by an *F* test. The significance of the effect of each variable on enzyme production was measured using a t test. The data were interpreted to obtain the response surface in the form of contours and 3D images showing the interaction of the factors.

### Partial purification of *β*-glucosidase

#### Ammonium sulfate precipitation and dialysis

Salting out with ammonium sulfate was used as the first step in protein purification. In this study, 30, 40, 50, 60 and 70 % ammonium sulfate was used for protein precipitation.

After dissolution of the salt, the solutions were centrifuged at 10,000 rpm for 10 min at 4 °C. The precipitate was dissolved in 0.1 M phosphate buffer (pH 7.0). Dialysis was carried out using the ammonium sulfate precipitated sample. Samples were dialysed in 0.1 M phosphate buffer (pH 7.0) for 2 days in a cold room, with continuous stirring. The buffer was frequently changed after 24 h. After this, the sample was collected and stored in the freezer for further use.

#### Gel filtration chromatography

Gel filtration chromatography was carried out to separate the protein on the basis of size. Samples were added to an approximately 3.5 inch Sephadex-50 column. 35 fractions of the mobile phase were collected and all the fractions were checked for the presence of protein in a spectrophotometer at a wavelength of 280 nm. *β*-glucosidase assay was performed on the fractions which tested positive for the presence of protein.

## SDS-PAGE

SDS-PAGE (12 %) was carried out according to the procedure described by Laemmli (1970). Staining of the gel was done using both Coomassie Brilliant Blue as well as silver staining (Morrissey 1981). The approximate molecular weight of the enzyme was determined from the bands developed in the gel using standard protein marker.

### *β*-glucosidase characterization

The specific activity of the enzyme was checked at temperatures 4, 30, 37, 45 and 55 °C. The standard enzyme assay was carried out to quantify the enzyme activity. Similarly, the activity of the enzyme was also checked at pH 4, 5, 6, 7, 8, 9 and 10, to find out the pH in which the enzyme works best in. The standard enzyme assay was done to check the same. Enzyme kinetics study was done to understand the catalytic behaviour of the enzyme. 1 ml of enzyme was added to different concentration of substrate and the activity was checked using the standard assay at 410 nm. Value of *V*_max_ and *K*_*m*_ was calculated from the graph plotted against substrate concentration and enzyme activity.

#### Back propagation neural network (bpnn)

Back propagation networks were poised of layers of neurons. Each layer consists of neurons. The input and output layers were connected. The BPNN consists of an input layer, one or two hidden layers, and an output layer. A BPNN has* n* input nodes,* r* output nodes and a single hidden layer of* m* nodes. All the connections have multiplying weights associated with them. Therefore, the output *o*_*k*_ can be expressed as:

1 $${o_k}\left( x \right) = \sum\limits_{j = 1}^m {w{2_{kj}}.f\left( {\sum\limits_{i = 1}^n {W{1_{ji}}{x_i} + b{1_j}} } \right) + b{2_k}}$$

where function *f* is the transfer function or activation function, *W*1_*ji*_ is the weight between the* i*th input node and* j*th hidden, *W*2_*kj*_ is the weight between the * j*th hidden node and* k*th output node, *b*1_*j*_ is the bias at* j*th hidden node, and *b*2_*k*_ is the bias at* k*th output node.

In Eq. (1), function is a kind of mapping rule to convert neuron weighted input to output and also a kind of design to commence non-linear influence into the BPNN. The sigmoid function is used to process the network. Our method is based on the same protocol of the current sigmoid function and is shown as

2 $$f\left( x \right) = \frac{1}{{1 + e^{ - x} }}$$

In BPNN, each unit within the same layer is not connected to one another, and the connection between the adjacent layers is expressed based on the weighted coefficient. Then the difference between the expected output and actual output is used as adjusting parameter for weighted coefficient. The process is repeated until the difference is minimum. The optimum parameter is obtained through adjusting the weighed values in the neural network, and optimum means that the squared deviation between the network output *o*_*k*_ and the actual values as target values *t*_*k*_’s achieve a minimum, i.e.,

3 $$E = \frac{1}{2}\sum\limits_{k = 1}^{r} {\left( {o_{k} - t_{k} } \right)^{2} }$$

The whole process is continued for each of the study cases, then back to the first case again, and so on. The cycle is repeated until the overall error value drops below some predetermined threshold. At this point, we say that the network has learnt the problem “well enough”—the network will never exactly learn the ideal function, but rather it will asymptotically approach the ideal function.

## Results

14 isolates were obtained from selfish wastes and further screened for the production of *β*-glucosidase enzyme which has best applications in production of bioethanol. Out of 14 isolates, the isolate named VIT117 produced maximum amount of *β*-glucosidase (1.56 U/ml) and this isolate was used for further studies. The organism was found to be gram-negative rods with motility. Biochemical tests were performed for the isolate VIT117 and the results were tabulated in Table 1. It was found that the organism utilizes almost all carbon sources used in the study. A growth curve was plotted, from which it was seen that the organism shifts from the lag phase to the log phase in 4–8 h, and shifted to stationary phase after 48 h of inoculation (Supplementary Fig. 1).

**Table 1:** Biochemical tests for the isolate

S. No.	Test	Result
1	Citrate utilization	Positive
2	Lysine utilization	Positive
3	Ornithine utilization	Positive
4	Urease utilization	Positive
5	Plenylalanine utilization	Negative
6	Nitrate reduction	Negative
7	H_2_S production	Negative
8	Glucose utilization	Positive
9	Adonitol utilization	Positive
10	Lactose utilization	Positive
11	Arabinose utilization	Positive
12	Sorbitol utilization	Positive

## 16 s rRNA sequencing

Isolate VIT117 which was able to produce significant amount of *β*-glucosidase was given for 16 s rRNA sequencing to identify the maximum matching organism. The sequence on blasting was found to have 99 % sequence similarity with *Proteus mirabilis*. Phylogenetic tree construction was done which identified the isolate as *Proteus mirabilis* and the organism was named as *Proteus mirabilis* VIT117 (Supplementary Fig. 2).

### Effect of various parameters on *β*-glucosidase production

**Fig. 1:**
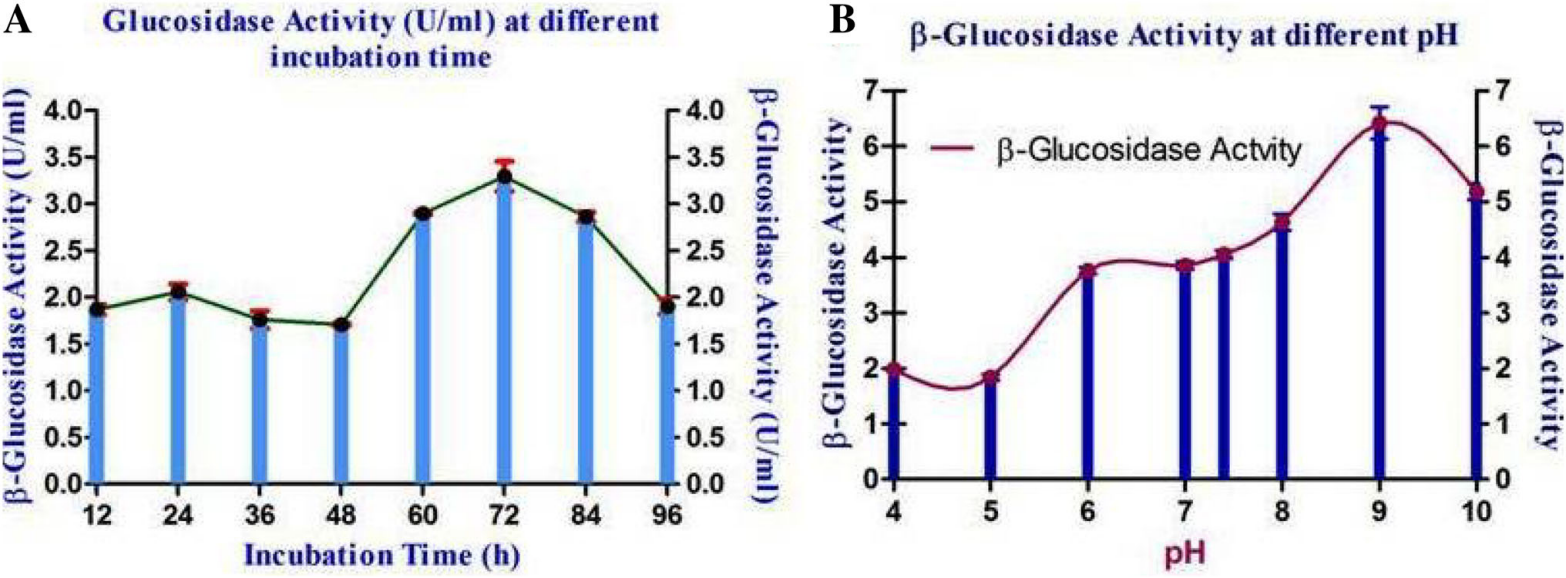
*β*-Glucosidase activity of *Proteus mirabilis* VIT117 at different incubation time (**a**) and pH (**b**)

**Fig. 2:**
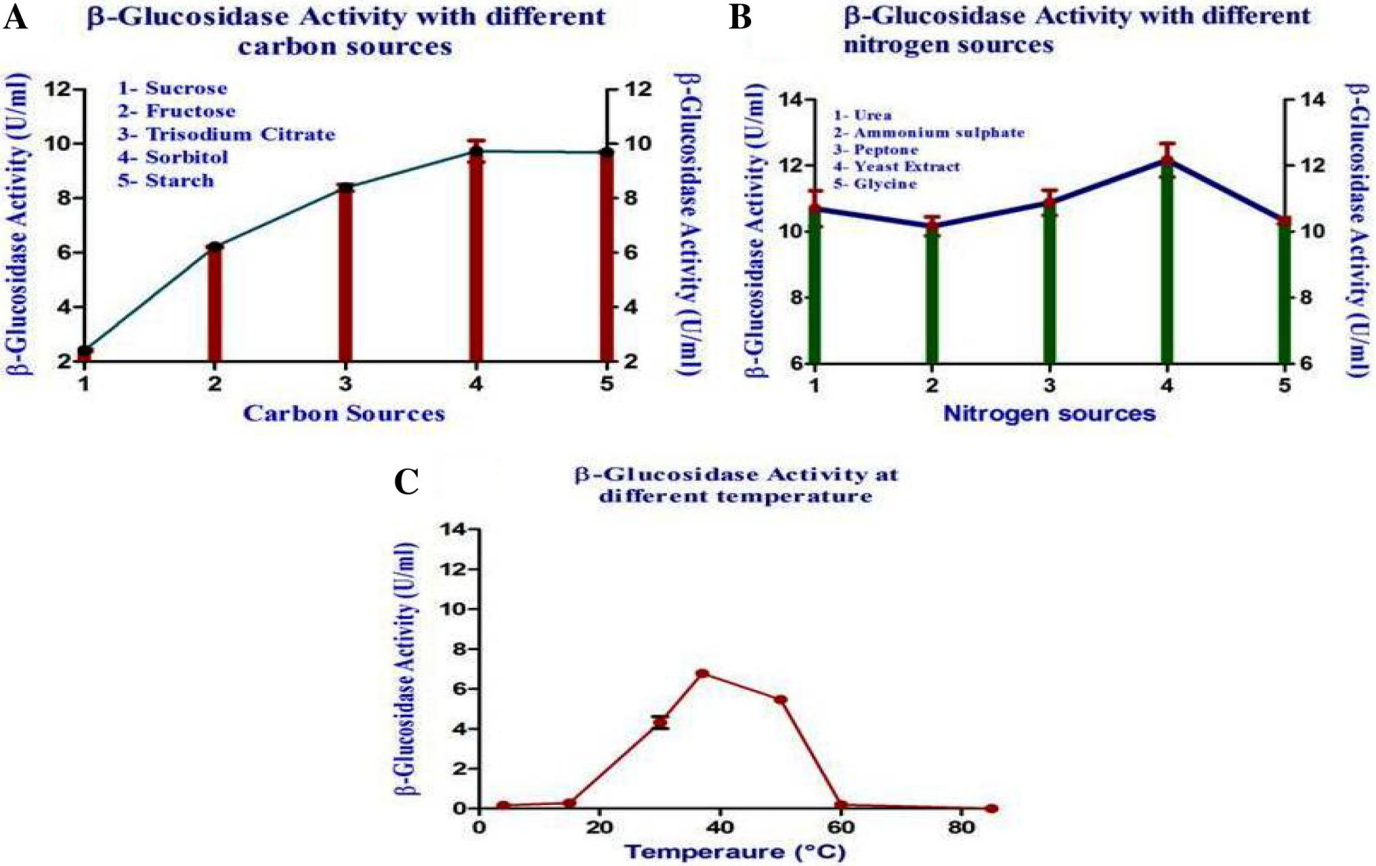
*β*-Glucosidase activity of *Proteus mirabilis* VIT117 for different carbon sources (**a**), nitrogen sources (**b**) and temperatures (**c**)

72 h or a period of approximately 3 days incubation time was found to be optimum with a maximum enzyme production of 3.45 U/ml (Fig. 1a). Hence, it can be concluded that the enzyme was being produced in the stationary phase. Maximum enzyme activity of 6.71 U/ml was recorded at pH 9 for an incubation time of 72 h (Fig. 1b). 37 °C was found to be the optimum temperature for the isolate to produce *β*-glucosidase. The enzyme activity recorded when the culture was maintained at pH 9 and incubated for 72 h at 37 °C was 6.79 U/ml (Fig. 2c). Sorbitol was found to be the best carbon source which recorded a maximum enzyme activity of 10.12 U/ml at pH 9 with 72 h incubation time at 37 °C (Fig. 2a). Yeast extract was found to be the best nitrogen source that had a maximum enzyme activity of 12.67 U/ml when pH was maintained at 9, temperature at 37 °C with incubation time of 72 h (Fig. 2b). It was determined after experimentation, that 1 % (w/v) sorbitol, 1 % (w/v) yeast extract, coupled with pH 9, incubation time of 3 days and incubation temperature of 37 °C greatly increases the growth of the microbes, thereby increasing the *β*-glucosidase production as well (14.58 U/ml).

### Optimization by Plackett–Burman

**Fig. 3:**
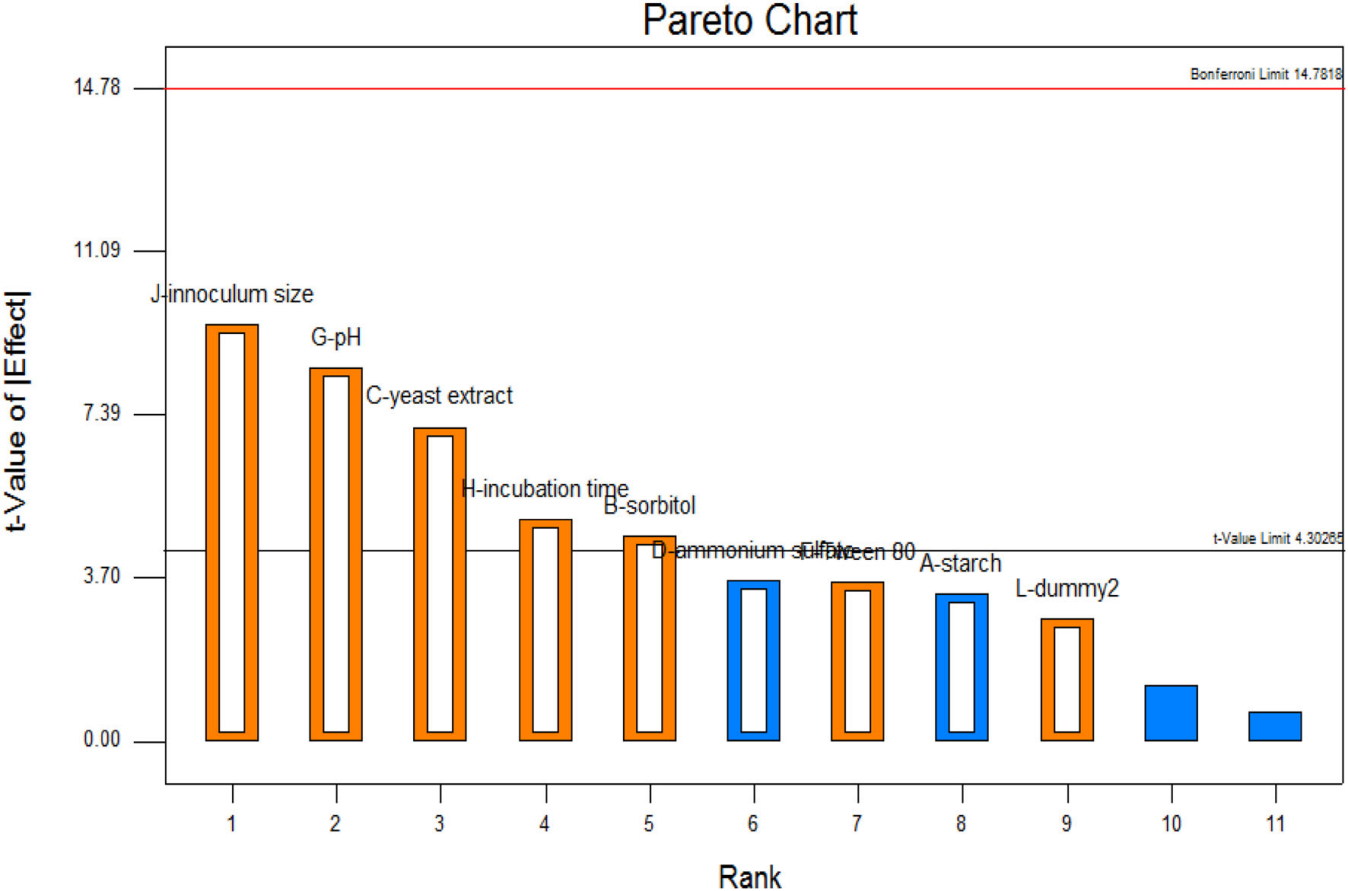
Pareto chart for significance of the variable

From the Plackett–Burman design, the factors that were found to be the most significant in the enhancement of *β*-glucosidase production are inoculum size, pH, yeast extract, incubation time and sorbitol, illustrated clearly in the Pareto chart (Fig. 3). The *R* ^2^ value was found to be 0.9934, indicating that up to 99.34 % of the data variability could be explained by the model.

### Response surface method (RSM)

**Fig. 4:**
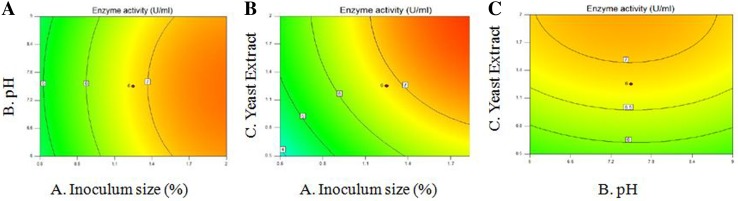
Three-dimensional response surface plot of variables (**a**) pH and inoculum size and its effect on (**b**) inoculum and yeast extract and its effect on β-glucosidase production (**c**) yeast extract and pH and its effect on β-glucosidase production

The optimum concentrations of the significant variables were found to be 2 %, 9 and 2 %, respectively, with the maximum enzyme activity of 16.42 U/ml. The contour plots seen in the three dimensional plot were observed to be elliptical in shape, meaning that all the parameters interacted with each other and are dependent on one another, (Fig. 4a–c). The statistical significance of the model was evaluated by F-test analysis of variance (ANOVA). Additionally, the probability value of 0.0001 for the model revealed that this regression was significant. Also, the influence by inoculum size, pH and yeast extract on *β*-glucosidase production was found to be statistically significant (Table 2).

**Table 2:** ANOVA table for response surface methodology

Source	*p* value Prob > *F*
Model	<0.0001
A-inoculum size	<0.0001
B-pH	0.9909
C-yeast extract	0.0003
AB	0.8911
AC	0.8911
BC	0.8685
A^2^	0.0010
B^2^	0.0584
C^2^	0.0366

### Artificial neural networks

In this study, the process parameters like inoculum size, pH and yeast extract were taken as the inputs and activity of the enzyme is taken as output for analysis by artificial neural network model. Out of the 20 experimental data points, twelve were used as training data sets, four for validating and the other four for testing the neural network. Initially, the ANN was trained to reach the error goal of 0.1. The performance of the neural network model was studied with special attention to their generalization ability and training time. For the best performance of the BPNN, the proper number of nodes in the single hidden layer or double hidden layers was selected through a trial and error method based on mean square error (MSE) values. The experimental response, RSM response (calculated from the quadratic equation given below) and ANN response were compared in Table 3. The maximum absolute error for ANN prediction when compared to RSM was 11.80 %. Up to 30 % error was allowed for ANN prediction and we got less error in percentage. Based on the absolute error results, BPNN proved that it will be used in prediction with bulk data instead of RSM or manual experimental assays.

**Table 3:** Experimental response vs RSM and ANN prediction

Run	Inoculum	pH	YE	Experimental Response (U/ml)	RSM Response (U/ml)	ANN Response (U/ml)	Error b/w RSM and ANN	Absolute error (%)
1	2	6	2	14.42	15.24	15.54	0.30	1.93
2	0.5	6	0.5	7.80	7.08	7.90	0.82	10.37
3	2	9	0.5	11.82	12.60	12.82	0.22	1.72
4	1.25	7.5	2.51	14.24	14.30	14.40	0.10	0.69
5	2	6	0.5	11.78	12.54	12.62	0.08	0.63
6	0	7.5	1.25	4.28	5.90	6.42	0.52	8.09
7	1.25	10	1.25	12.24	12.14	12.46	0.32	2.56
8	0.5	9	0.5	7.82	7.32	8.30	0.98	11.80
9	0.5	6	2	10.82	10.14	10.24	0.10	0.97
10	1.25	7.5	1.25	13.60	13.64	13.82	0.18	1.30
11	1.25	5	1.25	12.00	12.08	12.30	0.22	1.78
12	1.25	7.5	1.25	13.6	13.64	14.24	0.6	4.21
13	2.51	7.5	1.25	14.82	15.42	15.64	0.22	1.41
14	1.25	7.5	1.25	13.60	13.64	14.20	0.56	3.94
15	1.25	7.5	0	9.60	9.50	9.62	0.12	1.24
16	0.5	9	2	10.82	10.16	10.30	0.14	1.35
17	1.25	7.5	1.25	13.60	13.64	13.82	0.18	1.30
18	1.25	7.5	1.25	13.60	13.64	14.34	0.70	4.88
19	2	9	2	16.42	14.76	15.16	0.4	2.63
20	1.25	7.5	1.25	13.60	13.64	14.34	0.70	4.88

$$\begin{aligned} {\text{Enzyme activity }} = - 6.32481 + 4.56769*{\text{Inoculum size }} + 1.86318*{\text{pH}} + 2.55325*{\text{Yeast extract}}{-} \hfill \\ \, 0.021222*{\text{ Inoculum size}}*{\text{pH}}{-}0.042444*{\text{Inoculum size}}*{\text{Yeast extract}}{-}0.025667*{\text{pH}}*{\text{Yeast}} \hfill \\ {\text{ extract }}{-}1.03941*{\text{ Inoculum size}}^{2} {-} \, 0.12024*{\text{pH}}^{2} {-}0.54317*{\text{Yeast extract}}^{2} \hfill \\ \end{aligned}$$

### Partial purification of protein

**Fig. 5:**
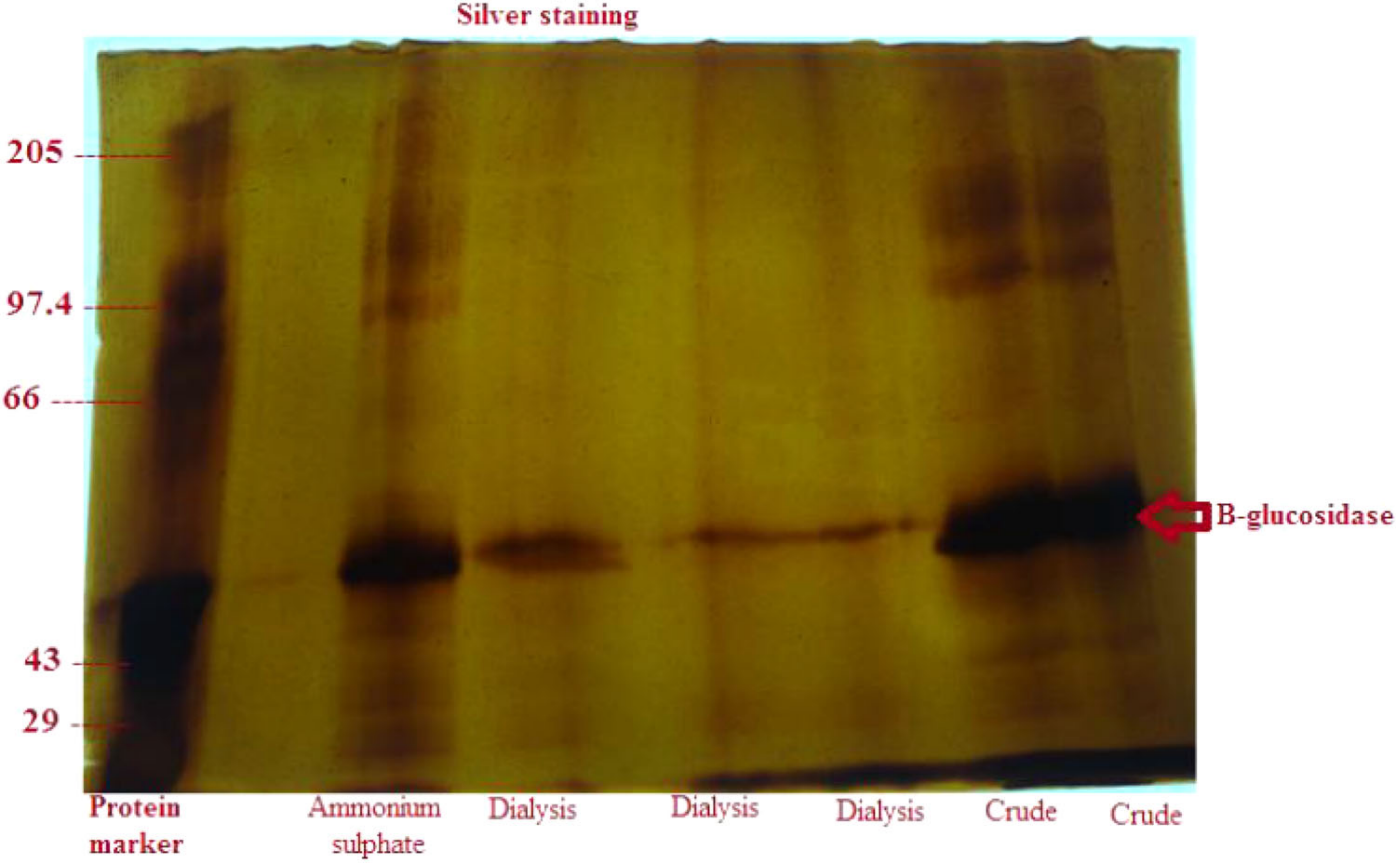
Silver staining

Ammonium sulfate precipitation, dialysis and column chromatography were done to purify *β*-glucosidase which has many applications. The individual fractions like ammonium sulfate precipitated phase, dialyzed fraction, were loaded in the SDS-PAGE gel along with the crude sample and a protein marker to visualize the bands of protein obtained (Fig. 5). Dialyzed sample was loaded in column chromatography to purify *β*-glucosidase fraction for further studies. 35 fractions were collected where the 7th fraction shows positive *β*-glucosidase activity. The purified sample was loaded in the SDS-PAGE gel to elucidate the molecular weight. Molecular weight of the enzyme was also predicted to be close to 50 kDa in reference to the protein marker.

### Enzyme characterization

**Fig. 6:**
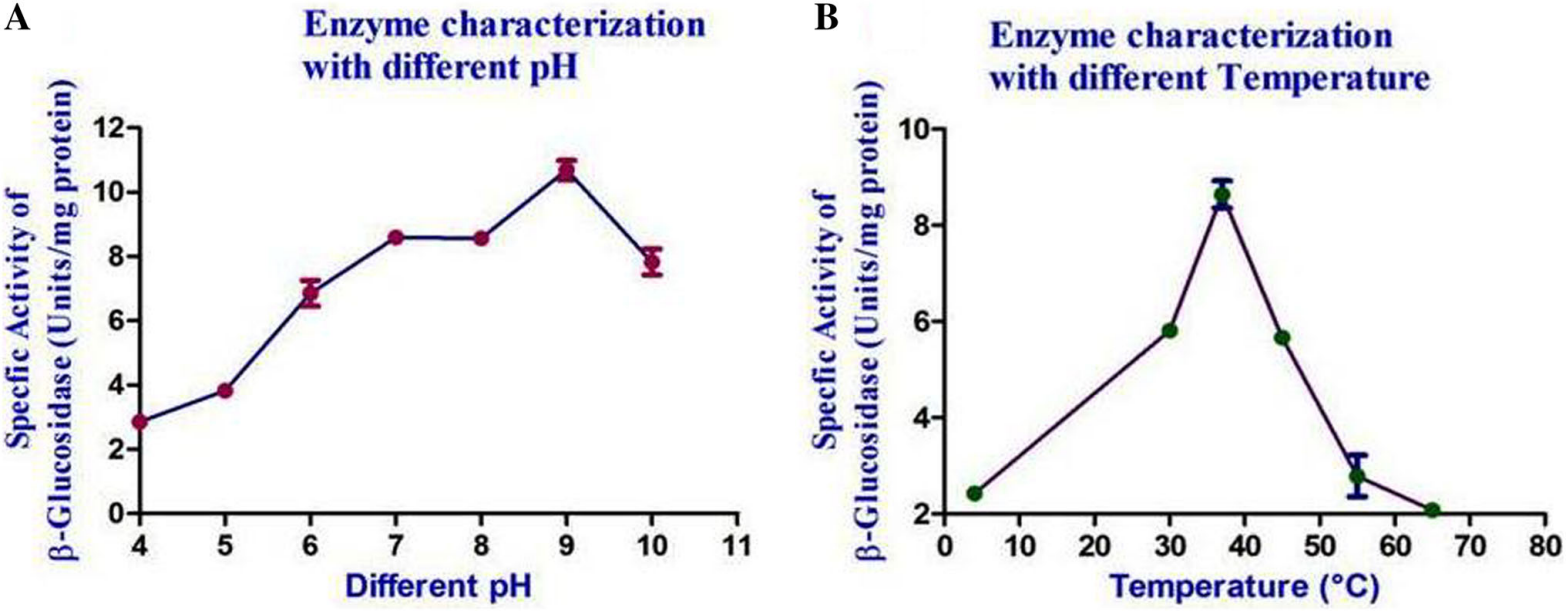
Partially purified *β*-glucosidase enzyme characterization with different pH (**a**) and temperatures (**b**)

From enzyme kinetics study conducted, the Michaelis–Menten parameters *V*_max_ and *K*_*m*_ were determined; it was found that our *β*-glucosidase had 5.613 U/ml and 0.082 mM as *V*_max_ and *K*_*m*_ values, respectively. The partially purified enzyme was characterized for pH and temperature to elucidate the optimum activity of the enzyme. pH 9 was found to be the optimum pH with an enzyme specific activity of 10.987 U/mg (Fig. 6a). 37 °C was found to be the optimum temperature with enzyme specific activity of 8.921 U/mg (Fig. 6b).

## Discussion

The main objective of this project was to screen, optimize and characterize *β*-glucosidase enzyme from bacteria obtained from the shells of prawns. Such a source was chosen because prawn shells are made of chitin, which is a long-chain polymer of *N*-acetylglucosamine, a derivative of glucose. The structure of chitin is comparable to the polysaccharide cellulose. These units form covalent *β*-1,4 linkages, and hence, can be acted upon by the enzyme of interest, *β*-glucosidase (Bagudo et al. 2014). The bacteria isolated from the prawn shells were further checked to see if they can utilize chitin. The results were positive, as our organism *Proteus mirabilis* VIT117 was growing successfully in chitin agar without producing chitinase. In the study conducted by Bohlin et al. (2010), eight different fungal species were found to produce the fungal *β*-glucosidase rather chitinase to utilize chitin agar. This concluded that the organism was able to produce *β*-glucosidase enzyme, and further tests were then carried out to determine the factors which contributed to the significant enhancement of the enzyme production. The assay for *β*-glucosidase tested positive only after an approximate incubation time of 72 h, which corresponds to the stationary phase of the growth curve. Hence, it can be deduced that the enzyme was being produced during the stationary phase of the *Proteus mirabilis* VIT 117 life cycle.

Classical method of optimization was carried out with reference to Dekker (1986), to find the parameters that provided the bacteria with optimum conditions for growth. Plackett–Burman design was implemented for a total of 11 factors, whose effects on the microbial growth and production of β-glucosidase were studied, with reference to the study conducted by Job et al. (2010). The interaction of the most significant factors was further studied in RSM by referring to the similar work carried out by Jiang-Ning et al. (2008). The 3D plots obtained showed that the factors interacted with one another and that they were dependant on each other.

Separate bands of proteins were obtained after the electrophoresis of the purified enzyme and the molecular weight of the *β*-glucosidase was estimated to be close to 50 kDa in reference to the protein marker. A similar study was carried out by Painbeni et al. (1992), where the molecular weight of *β*-glucosidase obtained from *Bacillus polymyxa* was also found to be 50 kDa.

In the study conducted by Bohlin et al. (2010), with eight different fungal species the *K*_*m*_ for fungal *β*-glucosidase varied by a factor of 3 with the lowest value determined for *C*. *globosum* (0.95 mM). But in our study the *V*_max_ and *K*_*m*_ values were found to be 5.613 and 0.082 mM indicating that bacterial *β*-glucosidase has more affinity towards substrate when compared with fungal *β*-glucosidase.

### Conclusion

Marine microorganisms take an active part in the mineralization of complex organic compounds (Kumar et al. 2008). Their participation in the degradation of organic compounds and retting of ropes and fibers testifies to their potential as a rich source of hydrolytic enzymes of industrial importance. In this context, in the present study, an attempt was made to isolate a potent *β*-glucosidase-producing bacterium from the marine environment. *β*-glucosidase exhibits wide substrate specificity and hence its field of applications is also wide. The production of ethanol from lignocellulosic residues is considered to be one of the major applications of *β*-glucosidase (Korotkova et al. 2009). The data obtained from this study can be utilized for mass production of the enzyme in the industry and may help to ease the process of bioethanol production from lignocellulosic feedstock. Ethanol produced from biomass is a clean fuel that is the most widely used biofuel when blended with gasoline.

## Electronic supplementary material

Below is the link to the electronic supplementary material.

Supplementary material 1 (DOCX 103 kb)
