# Supplementary figures and images for: Screening, production, optimization and characterization of β-glucosidase using microbes from shellfish waste
Source: 3 Biotech. 2016 Oct 3;6(2):213. doi: 10.1007/s13205-016-0530-7 (PMC5047857; doi:10.1007/s13205-016-0530-7)

Figure 1: Growth curve of the isolate VIT117


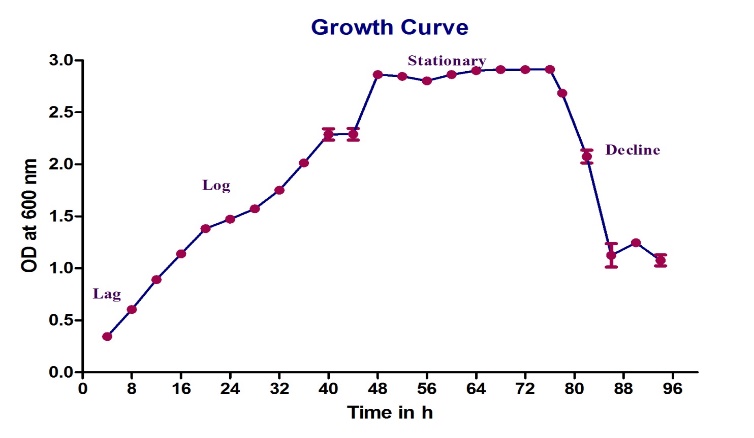


Figure 2: Phylogenetic tree for the isolate VIT117


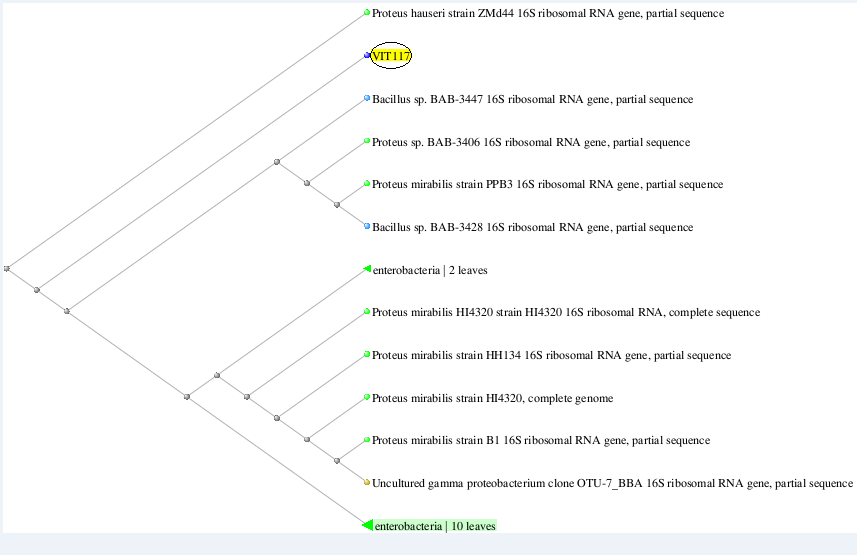

Supplement: Supplementary file 1 — Supplementary material 1 (DOCX 103 kb) [file 13205_2016_530_MOESM1_ESM.docx]
